# Clinical performance of different types of posterior bulk-fill resin composite restorations in permanent teeth: a systematic review

**DOI:** 10.1186/s12903-026-10017-x

**Published:** 2026-10-26

**Authors:** Asmaa S. Elsaeed, Youniss Saleh Harp, Hamdi Hamama, Salah H. Mahmoud

**Affiliations:** 1 Conservative Dentistry Department, Faculty of Dentistry, Mansoura University, Mansoura, 35516 Egypt; 2 Conservative Dentistry Department, Faculty of Oral and Dental Medicine, Delta University for Science and Technology, Gamasa, Egypt

**Keywords:** Bulk-fill composite, Pre-heating, Flowable bulk-fill, Sonicfill composite, Thermoviscous composite, Systematic review

## Abstract

**Background:**

Bulk-fill resin composites were developed to overcome limitations of incremental placement techniques: time consumption and air bubble entrapment. They are available in different viscosities, including high-viscosity (sculptable) and low-viscosity (flowable). Furthermore, practitioners deploy various techniques, such as sonic activation and preheating, to reduce the viscosity of bulk-fill resin composites. However, few studies have evaluated the clinical performance of different bulk-fill resin composite types. Consequently, this systematic review aimed to evaluate and compare the clinical performance of sonic-activated, preheated, and flowable bulk-fill resin composites versus conventional high-viscosity bulk-fill materials in permanent posterior teeth, regarding the following parameters: functional (retention, marginal adaptation, and anatomic form), esthetic (marginal discoloration, color match, and surface texture), and biological parameters (secondary caries and postoperative sensitivity).

**Methods:**

This systematic review was conducted in accordance with PRISMA guidelines. An electronic search of PubMed and ScienceDirect identified clinical trials published between 2015 and 2025 that compared different types of bulk-fill resin composites. Clinical studies evaluated any cavity class in permanent posterior teeth with at least a one-year of follow-up. Study selection and data extraction were performed independently by two reviewers using predefined inclusion and exclusion criteria and a standardized, pilot-tested data extraction form. Included variables were study characteristics, sample size, type of restorative material, evaluation criteria, follow-up duration, and clinical outcomes. The modified Cochrane Risk of Bias Tool was used to assess the quality of the selected studies.

**Results:**

Out of 1041 records, 33 full-text articles were assessed for eligibility, with six randomized controlled trials (RCTs) finally included. Overall, no significant differences were observed among bulk-fill resin composite types regarding retention, surface texture, anatomic form, secondary caries, or postoperative sensitivity. Some trends suggested slightly better marginal adaptation for flowable or preheated composites. Additionally, one included study reported no significant differences between Class I and Class II restorations.

**Conclusions:**

Different types of bulk-fill resin composites perform similarly for most functional and biological parameters, with marginal adaptation and discoloration being the parameters requiring further long-term investigation.

**Supplementary Information:**

The online version contains supplementary material available at https://doi.org/10.1186/s12903-026-10017-x.

## Background

Resin composite restorations widely used for the treatment of posterior lesions [1]. However, polymerization shrinkage and stresses remain a major challenge. These stresses at tooth-restoration interface may lead to microleakage, postoperative sensitivity, and eventual restoration failure [2]. Nevertheless, incremental layering techniques were introduced to minimize the negative impact of polymerization stresses. They are time-consuming and may result in voids between successively built layers [3–5]. 

Bulk-fill resin composites were developed to overcome limitations associated with incremental placement. These materials can be placed in a single increment of up to 4–5 mm [6]. This is facilitated by greater translucency and more efficient photoinitiators that ensure adequate depth of cure and reduced shrinkage [7, 8]. Moreover, modified monomer systems have been introduced to control the polymerization process and minimize shrinkage stress.

Bulk-fill resin composites are available in different viscosities: low-viscosity (flowable), high-viscosity (sculptable), and modified high-viscosity bulk-fill resin composites such as sonic-activated or preheated composites. These new generations of bulk-fill composites enhance handling characteristics and internal adaptation to cavity walls.

Compared with conventional nanohybrid resin composites, low-viscosity bulk-fill resin composites exhibited reduced mechanical properties and superior marginal adaptation due to their lower filler content [8–11]. Conversely, high-viscosity bulk-fill resin composites demonstrate improved mechanical properties owing to higher filler loading; however, they may present challenges in marginal and internal adaptation [9, 11]. Sonic-activated bulk-fill resin composites have been reported to enhance adaptation through a temporary reduction in viscosity induced by sonic energy [12, 13]. Similarly, preheating has been suggested to improve composite flow, thereby enhancing adaptation and reducing film thickness, as reported in some studies [14, 15]. 

Viscosity variations among bulk-fill composite restorative materials affect restoration adaptation, handling properties, and Retrospective long-term survival rate. Clinical performance of bulk-fill resin composites has been compared with incrementally layered conventional resin in previous evidence-based dentistry studies [16–20]. Most studies have reported comparable outcomes. However, there is insufficient evidence comparing the clinical performance of different types of bulk-fill resin composites. Consequently, this systematic review aimed to evaluate and compare the clinical performance of sonic-activated, preheated, and flowable bulk-fill with that of conventional high-viscosity bulk-fill materials in permanent posterior teeth, regarding the following parameters: functional (retention, marginal adaptation, and anatomic form), esthetic (marginal discoloration, color match, and surface texture and biological (secondary caries and postoperative sensitivity).

## Methods

This systematic review was conducted in accordance with the Preferred Reporting Items for Systematic Reviews and Meta-Analyses (PRISMA) guidelines [21, 22]. This systematic review was registered in PROSPERO (International Prospective Register of Systematic Reviews) under number: CRD42025640119. The research question was formulated as follows: In permanent posterior teeth, how does the clinical performance of sonic-activated, preheated, and flowable bulk-fill resin composites compare with that of conventional high-viscosity bulk-fill composites in terms of functional, aesthetic, and biological outcomes? This question was structured according to the PICOS framework (Population, Intervention, Comparison, Outcomes, Study design) [23].

- P (Population): Participants with permanent upper or lower posterior teeth affected by simple carious lesions (involving a single tooth surface), compound carious lesions (involving two adjacent surfaces), or complex carious lesions (involving three or more surfaces), or non-carious cervical lesions (NCCLs), restored with direct resin composite restorations.
- I (Intervention): Posterior teeth restored with different types of bulk-fill (BF) composites, including sonic-activated BF, heated BF, and/or flowable BF composite.
- C (comparison): Posterior teeth restored with conventional high-viscosity bulk-fill composite.
- O (outcome): clinical performance of different types of bulk-fill composite restorations regarding, functional (retention, marginal adaptation, and anatomical form), esthetic (color match, marginal discoloration) aspects, and biological (post-operative sensitivity and secondary caries).
- S (Study design): Randomized clinical studies (RCTs), with follow-up.

The extracted clinical outcomes were categorized according to the FDI World Dental Federation (FDI) criteria domains:

- Functional domain: retention, marginal adaptation and anatomical form.
- Esthetic domain: color match and marginal discoloration.
- Biological domain: post-operative sensitivity, and secondary caries.

### Eligibility criteria

The following inclusion criteria were applied to the selected studies: randomized clinical trials (RCTs) comparing the clinical performance of different types of bulk-fill resin composites in cavities of any configuration (simple, compound, or complex) and in non-carious cervical lesions (NCCLs) in permanent dentition. Additionally, eligible studies were required to include at least two different bulk-fill resin composite categories. Furthermore, only clinical studies published within the previous ten years, studies written in English, parallel or split-mouth clinical trials, clinical studies with at least a one year of clinical follow-up, and adult male or female patients of any age group were included.

Studies excluded following careful assessment of their full-text articles included those that were published before 2015, non-English publications, or studies performed on endodontically treated teeth or teeth considered abnormal, including those with developmental anomalies, severe structural defects, fractures, or other conditions that could potentially affect restoration placement or clinical evaluation. Furthermore, studies evaluating indirect restoration, case reports, case studies, and pilot studies were excluded. Neither systematic reviews nor literature reviews were included in the current evidence-based study. Furthermore, clinical trials comparing bulk-fill resin composites with conventional resin composite and trials that evaluated only one category of bulk-fill resin composites, were also excluded.

### Data sources

The National Library of Medicine (MEDLINE/PubMed) and ScienceDirect databases were searched for published studies that evaluated the clinical performance of different types of bulk-fill resin composites published between January 2015 and January 2025. Additionally, the reference lists of the included studies were manually screened to identify other relevant studies that were not identified through the electronic database search. Then, the studies were manually verified to find other research that were not recognized using electronic databases search. The keywords used were as follows: (““Resin composite” AND “Bulk-fill composite” AND “Pre-heating” OR “Flowable bulk-fill” OR “Sonic fill composite” OR “thermoviscous composite” OR “FDI Criteria” OR “Modified USPHS”).

### Study selection

Studies were selected in three steps: title, abstract, and full-text analysis. The studies obtained through manual and electronic searches were collected. Each reviewer assessed the included studies against the review’s eligibility criteria, and consensus was reached on their inclusion. An ID that combined the of the first author’s name and year of publication was given to each eligible study.

### Data extraction and synthesis

Data were extracted and analyzed for eligibility and risk of bis by the first reviewer (A.S.). After the data were tabulated and organized, a second and third reviewer (H.H.and Y.S) confirmed the extracted data and risk of bias assessments. Any disagreements regarding data extraction or risk of bias were resolved through discussion, and if consensus was not achieved, a fourth reviewer (S.H.) was consulted to provide the final judgment. If there were any missing data in any included study, an email was sent to the corresponding author.

Meta-analysis was not performed in this study due to the significant heterogeneity among the included studies. The included studies exhibited considerable heterogeneity regarding study design, intervention type, follow-up duration, and outcome reporting, which impeded direct comparability. Moreover, the lack of complete numerical data in several studies hindered the calculation of effect sizes. In light of the above, conducting a meta-analysis would not have generated a reliable summary estimate.

### Risk of bias analysis in individual studies

Risk of bias of the included studies was evaluated via the modified Cochrane Collaboration Risk of Bias tool which contained five domains include selection bias (randomization, allocation concealment), performance bias (blinding of participants and operators), detection bias (blinding of outcome assessment), attrition bias (loss to follow-up and missing data / participants), and reporting bias (unclear withdrawals or reported outcomes). Bias was classified as high, low or unclear. A risk of bias summary and graph for the six included studies were generated using RevMan 5.4 (RevMan 5.4, The Nordic Cochrane Centre, The Cochrane Collaboration, Copenhagen, Denmark) [24]. 

Studies were considered to have a low risk of bias if they were evaluated as low risk of bias in all five main domains to be considered “low” risk of bias. A trial was considered to have a high risk of bias if at least one domain was evaluated as having a high risk of bias. A trial was an unclear risk of bias if one or more items were found to have unclear risk. Furthermore, publication bias was not formally assessed (e.g., using a funnel plot) due to the small number of included studies (*n* = 6), as such analyses are considered unreliable when fewer than ten studies are available.

## Results

### Search details

**Fig. 1:**
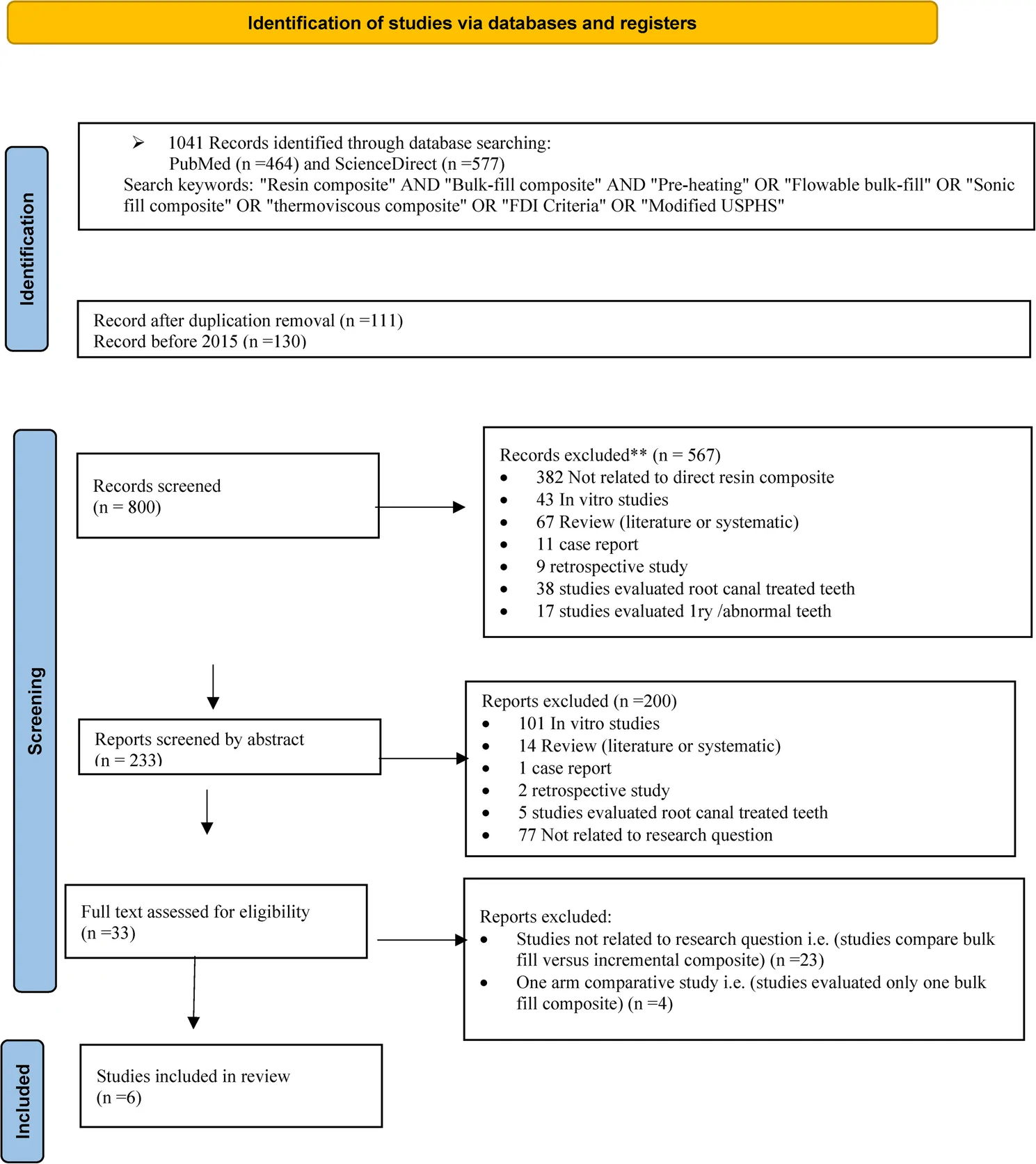
Flowchart demonstrating the study selection process based on the PRISMA statement

A total of 1,041 studies were identified through the search strategy. After removing duplicate using digital website and excluding studies published before 2015, 800 studies remained. After title screening, 567 records were excluded. Thus, 233 records were retained for further screening. After abstract screening, 200 studies were excluded. Following full-text assessment, 27 studies were excluded. Finally, six studies were included in this systematic review. The flowchart in Fig. 1 illustrates the study selection process.

### Risk of bias assessment

**Fig. 2:**
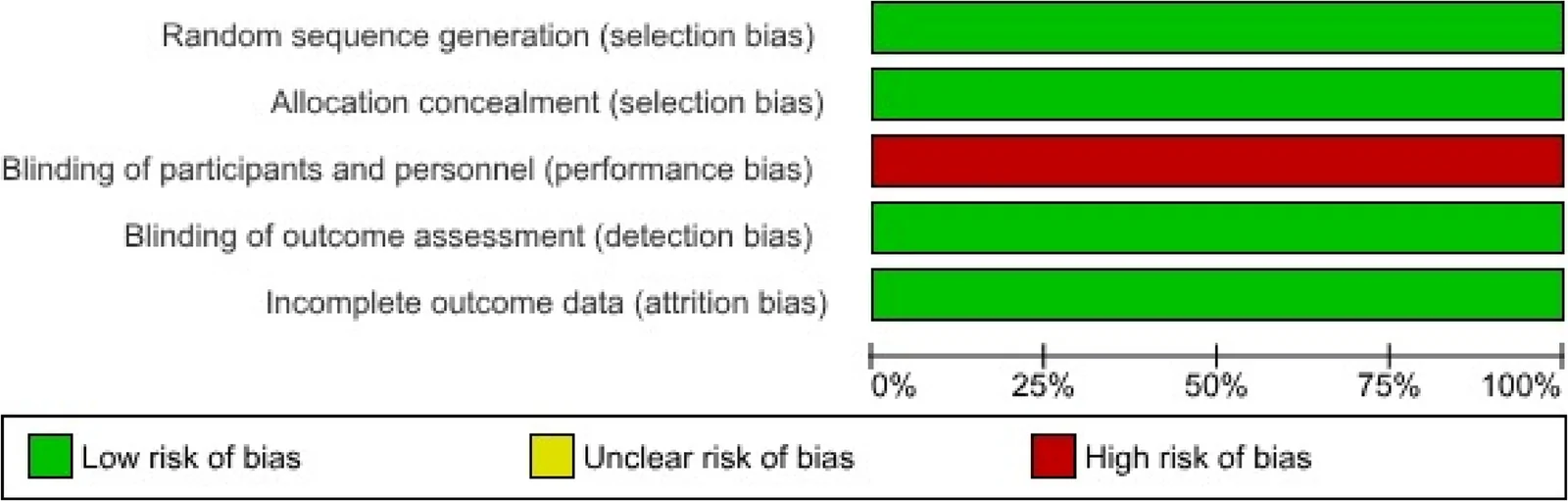
Risk of bias graph

**Fig. 3:**
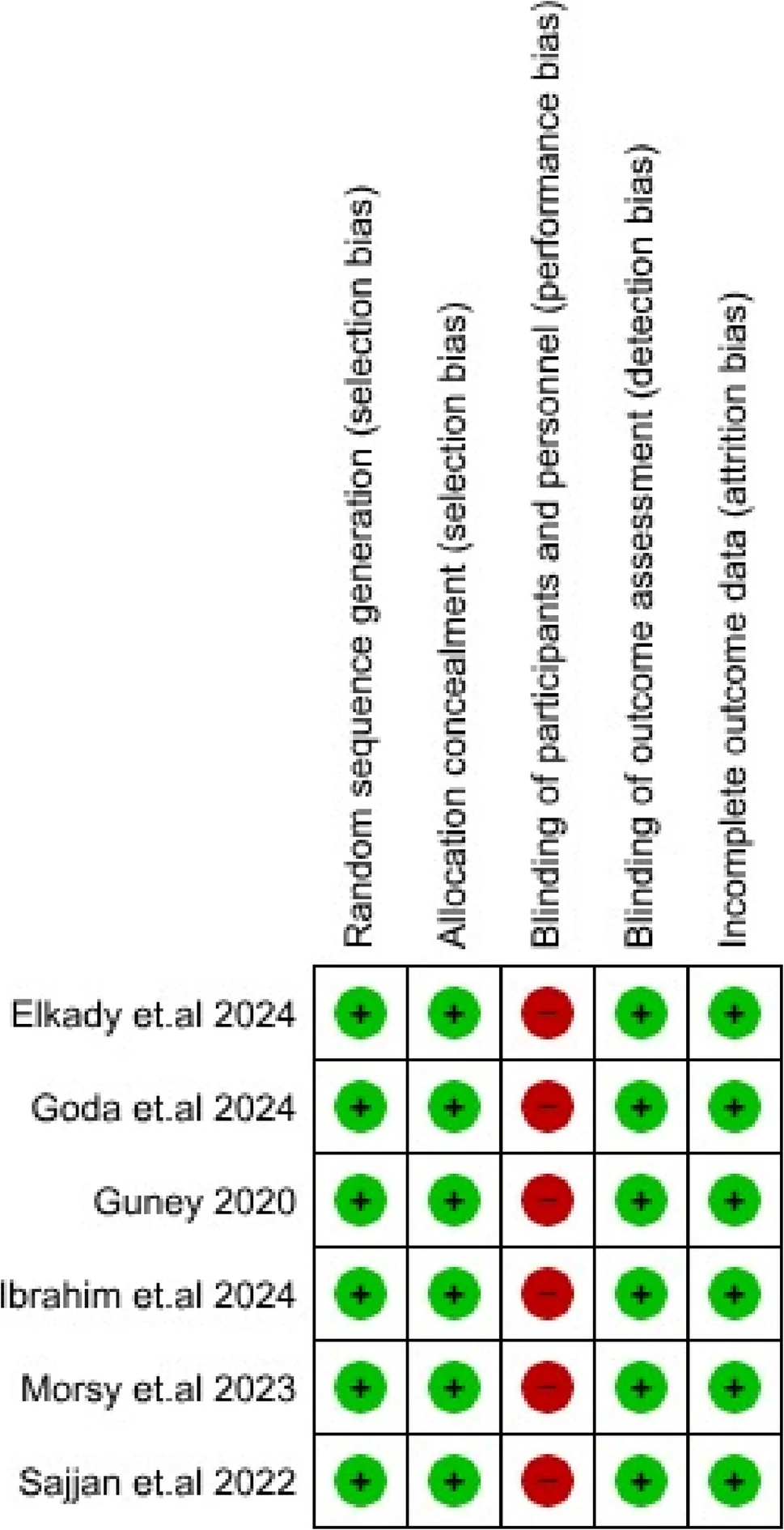
Summary of risk of bias assessment

All included studies were judged to have a low risk of bias in random sequence generation, allocation concealment, blinding of outcome assessment, and incomplete outcome data. However, all studies were rated at high risk of performance bias, as blinding of operators and participants to the restorative materials was not feasible. This consistent high risk of bias in the performance domain represents a significant methodological limitation of the included trials, and may influence the certainty of the overall findings. The randomization methods used in the included studies varied and included randomization Table [25] and online randomization website [26–28]. Additionally, Sajjan et al. [29]used computer-generated randomization method. While Morsy et al. [30] did not report the randomization method. The risk of bias assessment summary is presented in Table 1. The risk of bias graph and summary are shown in Figs. 2 and 3.

**Table 1:** Risk of bias assessment

Study ID	Random sequence generation (selection bias)	Allocation concealment (selection bias)	Blinding of participant and personnel (performance bias)	Blinding of outcome assessment (detection bias)	Incomplete outcome data (attrition bias)
Elkady et al [26] 2024	Low	Low	High	Low	Low
Guney and Yazici [25] 2020	Low	Low	High	Low	Low
Goda et al [28] 2024	Low	Low	High	Low	Low
Morsy et al [30] 2023	Low	Low	High	Low	Low
Ibrahim et al [27] 2024	Low	Low	High	Low	Low
Sajjan et al. [29] 2022	Low	Low	High	Low	Low

### Study characteristics

The selected studies were published between January 2015 and January 2025. The materials, objectives, and conclusions of each study are summarized in Table 2. The methodological assessment, including study design analysis, evaluation criteria, evaluation tools, sample size, restoration type, material selection, isolation method, follow-up, and recall rate are shown in Tables 3 and 4.

**Table 2:** Summary of studies included in systematic review

Study \ country	Year	Materials	Objective	Conclusion
(1)Elkady et al. [26] Egypt	2024	1-Filtek One Bulk (3 M Deutschland, Germany) - (Scotchbond Universal Plus (3 M Deutschland, Germany) 2–35% phosphoric acid (HV Bisco, USA)	To assess the clinical performance of class II restorations performed by repeatedly preheated resin composite “RC” at 68 ◦C up to ten times.	After 12-months follow-up, although no restoration needed replacement or repair in the 3 tested groups, restorations with single and ten times of preheating aided in better clinical performance of RC restorations compared to the non-preheated restorations. Preheating of RC for 10 times could be used safely with good clinical performance of restorations.
(2) Ibrahim et al. [27] Egypt	2024	1-X-tra Fil Bulk-fill posterior resin composite (VOCO GmbH, Cuxhaven, Germany) -Futurabond M+ Universal adhesive system (VOCO GmbH, Cuxhaven, Germany) 2-Eco-etch Acid etchant (Ivoclar Vivadent, Schaan, Liechtenstein)	To clinically evaluate the performance of non-preheated bulk fill resin composite in class II cavities versus one, five and ten-time preheating cycles at 68 °C.	The clinical performance of ten cycles of repeated preheated bulk fill resin composite was equivalent to that of non-preheating resin composites after 24 months follow-up period, with great improvement in manipulation.
(3) Goda et al. [28] Egypt	2024	1- Filtek™ BulkFill (3 M-ESPE, St.Paul, MN, USA) - Single Bond Universal (3 M-ESPE, St. Paul, MN, USA) 2- G-ænial™ BULK Injectable (GC, United Kingdom) - G-Premio BOND (GC, United Kingdom) 3-SonicFill3 (Kerr Corp, Orange, CA, USA) -OptiBond™ Universal (Kerr Corp, Orange, CA, USA) 4–37% phosphoric acid (N Etch, Ivoclar Vivadent),	To evaluate the clinical performance of bulk-fill resin composite systems with different viscosities in class II cavities	After 2 years of clinical follow-up, the bulk-fill composite systems with different viscosities seem to be esthetically, functionally, and biologically acceptable, with a promising clinical performance in class II cavities.
(4) Guney and Yazici [25] Turkey	2020	1-Tetric EvoCeram Bulk Fill (Ivoclar Vivadent, Schaan, Liechtenstein) -Excite F Dental adhesive (two-step etch-and-rinse) Ivoclar Vivadent 2-SureFil SDR Flow Bulk-fill Dentsply Caulk (Milford, DE, USA) 3-Ceram.X mono Nanoceramic (Dentsply Caulk) -Prime&Bond NT (two-step etchand-rinse) (Dentsply Caulk)	The objective of this study was to evaluate the 24-month clinical performance of different bulk-fill restorative resin materials in class II restorations.	All investigated bulk-fill restorations were found to be clinically acceptable and similar after 24 months of clinical follow-up Observation of a significant deterioration in marginal adaptation for all the restorations is another important outcome of the study.
(5) Morsy et al [30] Egypt	2023	1- VisCalor bulk (Voco GmbH, Cuxhaven, Germany) - Futurabond M+ (Universal adhesive) (Voco GmbH, Cuxhaven, Germany) 2-SonicFill 2 bulk fill composite (Kerr Corp, Orange, CA, USA) -Optibond All-In‐One (self-etch adhesive) (Kerr Corp, Orange, CA, USA) 3-Filtek Bulk Fill Posterior Restorative (3 M-ESPE, St.Paul, MN, USA) -Single Bond Universal Adhesive (3 M-ESPE, St.Paul, MN, USA) 4-Calcimol LC 5-Ionoseal (Resin modified glass-ionomer)	To evaluate the clinical performance of VisCalor and SonicFill versus the conventionally applied bulk fill composite restorations in Class I cavities over 18-month follow-up periods.	After 18-month follow-up all the tested materials exhibited good clinical behavior for the analyzed clinical criteria with the exception of marginal integrity. VisCalor bulk fill results were superior to those of SonicFill 2 and Filtek bulk fill
(6) Sajjan et al [29] India	2022	1-Tetric N-Ceram Bulk Fill (Ivoclar Vivadent products, Delhi, India) 2-Selective etch and rinse (N-Etch, Ivoclar Vivadent, Delhi, India) 3-N-Bond (Ivoclar Vivadent, Delhi, India)	The aim of this study was to assess the clinical behavior of bulk-fill composite resin restorations plasticized by preheating and ultrasonics.	The ultrasonics plasticization of composite resin demonstrated comparable results with that of preheated composite resin after 6- and 12-month recall.

**Table 3:** Methodological assessment of included studies

Study ID	Trial design	No of patients and gender	Number of restorations/ per group	Total number of restorations	Mean range of patients	Composite system		Placement technique	Evaluation criteria	Outcome evaluation	Follow-up period	Recall rate	Moisture control	Enamel bevel
						Control group	Intervention group/s							
(1) Goda et al. [28] Egypt	RCT Parallel	Total = 50 Male = 27 Female = 23	1-Filtek™ BulkFill = 20 2- G-ænial™ BULK = 20 3-SonicFill3 = 20 4-Preheated Filtek™=20	80	(18–35) years	-Filtek™ BulkFill (3 M-ESPE, St.Paul, MN, USA) - Single Bond Universal (3 M-ESPE, St. Paul, MN, USA)	1-G-ænial™ BULK Injectable (GC, United Kingdom) - G-Premio BOND (GC, United Kingdom) 2-SonicFill3 (Kerr Corp, Orange, CA, USA) -OptiBond™ Universal (Kerr Corp, Orange, CA, USA) 3- preheated Filtek™ BulkFill (3 M-ESPE, St.Paul, MN, USA) - Single Bond Universal (3 M-ESPE, St. Paul, MN, USA)	Single increment (4 mm)	FDI	color match, marginal adaptation, post- operative sensitivity, marginal discoloration, surface roughness, anatomical form, 2ry caries, Retention, periodontal response	2 Years	100%	Rubber dam	no
(2) Ibrahim et al [27] Egypt	RCT parallel	Total = 84 Male = 36 Female = 48	1-X-tra Fil Bulk-fill = 21 2-Preheated one X-tra Fil = 21 3-Preheated five X-tra Fil = 21 4-Preheated ten X-tra Fil = 21	84	(20–45) years	X-tra Fil Bulk-fill posterior resin composite (VOCO GmbH, Cuxhaven, Germany) -Futurabond M+ Universal adhesive system (VOCO GmbH, Cuxhaven, Germany)	1-preheated X-tra Fil (one, five and10) Bulk-fill posterior resin composite (VOCO GmbH, Cuxhaven, Germany) -Futurabond M+ Universal adhesive system (VOCO, GmbH, Cuxhaven, Germany)	Single increment (4 mm)	Modified USPHS	marginal adaptation, marginal discoloration, secondary caries, post- operative sensitivity, Retention, surface texture, , color match	2 Years	97.6%	Rubber dam	No
(3) Morsy et al [30] Egypt	RCT Split mouth	Total = 20 Male = nr Female = nr	1-Filtek Bulk Fill = 20 2-VisCalor bulk = 20 3-SonicFill 2 = 20	60	(25–40) years	Filtek Bulk Fill Posterior Restorative (3 M-ESPE, St.Paul, MN, USA) -Single Bond Universal Adhesive (3 M-ESPE, St.Paul, MN, USA	1- VisCalor bulk (Voco GmbH, Cuxhaven, Germany) - Futurabond M+ (Universal adhesive) (Voco GmbH, Cuxhaven, Germany) 2-SonicFill 2 bulk fill composite (Kerr Corp, Orange, CA, USA) -Optibond All-In‐One (self-etch adhesive) (Kerr Corp, Orange, CA, USA)	Single increment (4 mm)	Modified USPHS	secondary caries, postoperative sensitivity, color match, anatomic form, retention. marginal adaptation, marginal discoloration.	18 months	100%	Rubber dam	no
(4) Elkady et al [26] Egypt	RCT parallel	Total = 105 Male = 39 Female = 66	1-Filtek One =35 2-preheated one Filtek = 35 3-Preheated ten Filtek = 35	105	(18–40) years	-Filtek One Bulk (3 M Deutschland, Germany) - (Scotchbond Universal Plus (3 M Deutschland, Germany)	1- preheated one Filtek One Bulk (3 M Deutschland, Germany) 2-Preheated ten Filtek One Bulk (3 M Deutschland, Germany) - (Scotchbond Universal Plus (3 M Deutschland, Germany	multiple increments for all groups (2 mm)	Modified USPHS	retention and fracture, marginal discoloration, marginal adaptation, anatomic form, secondary caries, Color match.	1 Year	100%	Rubber dam	no
(5) Guney and Yazici [25] Turkey	RCT Split mouth	Total = 40 Male = 16 Female = 24	1-Tetric EvoCeram Bulk Fill = 40 2-SureFil SDR Flow Bulk-fill = 40	120	(20–41)	-Tetric EvoCeram Bulk Fill (Ivoclar Vivadent, Schaan, Liechtenstein) -Excite F Dental adhesive (two-step etch-and-rinse)	1-SureFil SDR Flow Bulk-fill Dentsply Caulk (Milford, DE, USA) -Ceram.X mono (Dentsply Caulk)	Single increment	Modified USPHS	Color match, marginal discoloration, marginal adaptation, anatomic form, secondary caries, retention and fracture	2 years	82.5%	Cotton roll and suction	no
(6) Sajjan et al [29] India	RCT Split mouth	Total = 28 Male = nr Female = nr	1-Preheated tetricN ceram = 28 2-Ultrasonic tetricN ceram = 28	56	18–45 years	No control group	1-(Tetric N-Ceram Bulk Fill) preheated in half teeth 2- Tetric N-Ceram Bulk Fill ultrasonic plasticization	Single increment	Modified USPHS	2ry caries, retention, marginal staining, postoperative sensitivity, marginal adaptation, and anatomic form.	1 year	75%	Rubber dam	no

*RCT*  Randomized clinical Trial, *NR*  not reported, *NCCLs*  Non-Carious cervical lesions, *USPHS* United States Public Health

**Table 4:** Methodological assessment of included studies

Study ID	Evaluation tools	Preheating and ultrasonic method	Randomization method	Most common arch/ teeth	Cavity classification	Cavity design and size	Number of Examiners	Number of operators
(1) Goda et al [28] Egypt	Digital photographs	-Therma-flo TM RC warming kit (Vista, Wisconsin, US) -SonicFill handpiece (speed setting 3)	Specific website	Not mentioned	Class II	Conservative	2	1
2) Ibrahim et al [27] Egypt	Mirror, probe and dental operating light	Therma-flo TM Composite Warming Device (VISTA APEX Inter-66 + + Med, Racine, USA)	Random Sequence Generator (https://www.random.org).	Equal no. in Upper/lower molars	Class II	Conservative	2	1
(3) Morsy et al [30] Egypt	SEM for evaluation of marginal integrity	1-VisCalor dispenser (programme1 setting) 2-SonicFill handpiece (speed setting 3)	Not mentioned	Not mentioned	Class I	Conventional	2	1
(4) Elkady et al [26] Egypt	Magnification of Loup and photographs	HeatSync RC warming kit (Bioclear, USA)	(https://www.random.org).	Maxillary premolars	Class II	Conservative	2	1
(5) Guney and Yazici [25] Turkey	Photographs and bitewing x-ray	Study not have preheated or sonic materials	table of random numbers	Mandibular premolars	Class II	Conservative	1	1
(6) Sajjan et al [29] India	Dental operating microscope	1-composite warmer (Delta Co., India) 2-modified 2-ultrasonic-retreatment tip-RT No 2 (Cricdental, India) in ultrasonic handpiece (Woodpecker, Guilin, China)	computer generated randomization (Research Randomizer)	Not mentioned	Class I or II	Conservative	NR	NR

### Evaluation of outcomes

Modified USPHS criteria were utilized in five of the included studies (83.3%) [25–27, 29, 30]. In contrast, the evaluation criteria used by Goda et al. [28] (16.6%) was the FDI. Evaluation aids varied among the included studies. In Ibrahim et al. [27] a mirror, probe and dental unit light were used as evaluation aids, while photographs were used for clinical assessment during all evaluation periods in three of the included studies [25, 26, 28]. Tooth replicas and scan electron microscopy were used to assess marginal integrity in one study [30]. Furthermore, a dental microscope and loupes were used in Sajjan et al. [29]

### Study designs

Included trials were randomized clinical trials (RCTs). Among the evaluated studies three (50%) used split-mouth designs [25, 29, 30]. while the remaining three studies (50%) [26–28] used a parallel design. The majority of the reviewed clinical trials (83.3%) reported the inclusion and exclusion criteria. However, Morsy et al. [30] did not report the inclusion and exclusion criteria. In addition, all six included studies indicated that they had received ethical approval from an appropriate institution. Furthermore, Participants in all included studies provided written informed consent [25–30]. Isolation was performed using rubber dam isolation in five of included studies [26–30] (83.3%). However, cotton rolls and saliva ejectors were used in only one study (16.6%) by Guney and Yazici [25]. 

### Evaluation of cavity preparation

Four (66.6%) of the included studies [25–28] evaluated the performance of bulk-fill resin composites in compound class II cavities. Morsy et al. [30]evaluated restorations in Black’s class I cavities. While Sajjan et al. [29]evaluated clinical performance of different bulk-fill types in class I and class II cavities. Enamel bevel and undercuts were not prepared in any of the included studies [25–30]. In all included studies, remaining soft carries were removed using large-sized round bur mounted in a low-speed handpiece or a sharp excavator. Cavity finishing to remove undermined enamel, eliminate surface roughness and round the internal line angles was preformed in all included studies.

### Interventions in included studies

Five (83.3%) of the included studies used conventional high-viscosity bulk-fill resin composite as a control group, whereas the intervention groups varied among all included studies. Among the five included studies, one study [28] compared conventional high-viscosity bulk-fill resin composite with three intervention groups (preheated, Sonicfill and injectable bulk-fill composites). Morsy et al. [30] evaluated conventional high-viscosity bulk-fill resin composite with two intervention groups (Sonicfill and preheated bulk-fill composites). Conventional high-viscosity bulk-fill resin composite was compared with flowable bulk-fill composites in the study by Guney and Yazici [25]. However, in two studies [26, 27], repeated preheating cycles were evaluated as intervention groups and compared with conventional high-viscosity bulk-fill composites. Furthermore, only one study [29] did not have a control group, instead comparing preheated and sonic-activated bulk-fill composites.

Different types of bulk-fill resin composite along with their manufacture-recommended dental adhesives were used in all included studies [25–30]. In three included studies [27, 28, 30] selective enamel etching was performed before the bonding step. However, in the remining two studies, one study [30] did not use a separate enamel etching step, whereas the other study [25] used total etching for enamel and dentin. Composite application was performed in a single increment up to 4 mm in all included studies. However, composite application was preformed incrementally (2-mm increments) in all evaluated groups in Elkady et al. [26]

### Evaluation during the follow-up period and recall rate

Follow-up duration and recall rates varied among the six included studies. Two-year follow-up was reported in Goda et al. [28], Ibrahim et al. [27] and Guney and Yazici [25] with recall rate 100%, 97.6% and 82.5% respectively. A one-year follow-up with recall rate 100% in Elkady et al. [26] and a one-year follow-up with recall rate 75% in Sajjan et al. [29]. A 18-month follow-up was reported in Morsy et al. [30] with 100% recall rate. Dropout were due to patient travel and the COVID-19 pandemic.

### Results of individual studies

#### Success rate assessment

It was reported in only one study [29]. In this study, only one bulk-fill resin composite restoration was plasticized either using preheating or ultrasonic activation. The overall success rate in the ultrasonic group was 100%; however, in the preheated bulk-fill composite group, it was 95.2%. No significant difference in success rate was recorded between the two different plasticizing modes (*p* > 0.05). The failure was attributed to the preheated bulk-fill composite group that had retention, postoperative sensitivity, and a marginal defect in one restoration.

#### Functional properties

##### Retention and fracture

Across the six included studies, retention was consistently reported as excellent for both conventional high-viscosity and various bulk-fill resin composite systems. Retention rates ranged from 95% to 100% (Alpha scores) across all follow-up periods. Statistical analyses in all studies revealed no significant differences between conventional high-viscosity bulk-fill and other bulk-fill resin composites, nor among the different types of bulk-fill materials (preheated, sonic-activated, or flowable) (*p* > 0.05).

In Goda et al.‘s study [28], all evaluated bulk-fill composite restorations (conventional high-viscosity, Sonicfill, preheated, and injectable) had an excellent retention rate of 100% over all follow up periods (*p* > 0.05). According to Morsy et al. [30], Elkady et al. [26] Guney and Yazic [25], and Ibrahim et al. [27] all tested bulk-fill composite restorations had a high retention rate with Alpha scoring in all follow-up periods (*p* > 0.05). In Sajjan et al. [29] study the retention rate was 100% and 95.2% for (sonic-activated) and (preheated) bulk-fill composites, respectively (*p* > 0.05).

##### Marginal adaptation

The marginal adaptation parameter was controversial, four of the included studies [25, 27–29] (66.6%) reported no significant differences between conventional (high-viscosity) and intervention bulk-fill resin composite systems. On the other hand, two studies [26, 30] (33.3%) showed that conventional high-viscosity bulk-fill resin composite systems exhibited significantly more marginal defects than the intervention group. However, no statistically significant differences were observed among the intervention groups (sonic, preheated, and /or flowable).

According to Goda et al. [28] and Ibrahim et al. [27], all restorations in the tested groups had excellent margins over a two-year follow-up period (100%, Alpha score). In the study by Guney and Yazici [25], four restorations received a Bravo score due to minor marginal discrepancy in both tested bulk-fill composite systems (conventional and flowable) at six months (*p* > 0.05). After two years of follow-up, the number of restorations with minor marginal discrepancy has increased to six in both groups (*p* > 0.05). The study by Sajjan et al. [29] study showed a “Charlie” score in only one restoration in preheated group after a 6-month follow-up with no significant difference compared with the sonic-activated group (*p* > 0.05).

Regarding Morsy et al. [30], all restorations retained an Alpha score during 12-month follow-up (*p* > 0.05). After 18 months, the conventional high-viscosity bulk-fill resin composite system demonstrated more pronounced marginal discrepancy compared to the preheated and sonicfill bulk-fill resin composite systems (*p* < 0.05). Furthermore, in Elkady et al. ‘s [26] study, significant marginal deficiency was recorded in the conventional high-viscosity bulk-fill composite system; only 20 restorations restored with conventional high-viscosity bulk-fill composite retained the Alpha rating (57.1%) compared with different preheating-cycle groups as all restorations (100%) retained Alpha rating (p < 0.05).

##### Anatomic form

Anatomic form was used as an evaluation item in all six included studies, and no significant differences were observed between conventional high-viscosity bulk-fill and all intervention bulk-fill resin composite systems. Furthermore, there were no significant differences between different bulk-fill resin composite types (*p* > 0.05).

In Goda et al. [28], Guney and Yazici [25], Elkady et al. [26], and Ibrahim et al. [27], all restorations retained Alpha scores with correct contour (100%, Alpha score) in all follow-up. In the study by Morsy et al. [30], only one restoration in the conventional high-viscosity group, 2 restorations in Sonicfill group, and no restorations in preheated group showed slight discount, after 18-months follow-up. The rates were 95% ,90%, and 100%, respectively (*p* > 0.05). In the study by Sajjan et al. [29], only one restoration had a Charlie score in the preheated group, with no significant difference compared with the sonic-activated group (*p* > 0.05). Detailed functional outcomes are summarized in Table 5.

**Table 5:** Synthesis matrix, statistical significance, consistency/ heterogeneity of functional outcomes

Outcomes	No. of Studies	Study (Author, Year)	Finding of each study	Summary finding	Statistical Significance	Consistency/ Heterogeneity
Retention	Six	1-Goda et al. [28] 2-Morsy et al. [30] 3-Guney & Yazıcı [25] 4-Sajjan et al. [29] 5-Elkady et al. 6-Ibrahim et al. [27]	Retention rates ranged from 95% to 100% (predominantly Alpha scores) across all studies	-All six studies reported excellent retention (95–100%) for both conventional and other bulk-fill composites types. -	No Significant differences among conventional, sonicfill, preheated, or flowable types (*p* > 0.05)	Consistent
Marginal adaptation	Six	1-Goda et al. [28] 2-Morsy et al. [30] 3-Guney & Yazıcı [25] 4-Sajjan et al. [29] 5-Elkady et al. 6-Ibrahim et al. [27]	1-All groups showed excellent margin (100%) over 2 years. 2-No significant differences at 6–12 months; after 18 months conventional high-viscosity bulk-fill composite showed more marginal defects 3-Minor marginal discrepancies noted in both tested materials. 4- only one restoration in preheated group showed Charlie score at six months 5-significant marginal deficiencies found in conventional high-viscous group compared to different preheated cycle groups 6-all restorations retained alpha score	-controversial results: ~70% reported [25, 27–29] no differences; others[26, 30] found conventional high-viscosity composites exhibited more marginal defects than preheated/sonic groups.	* Non-Significant (*p* > 0.05) for 4 studies [29] *Significant difference between conventional and other composite types in only 2 studies (*P* < 0.05)	Not Consistent
Anatomic form	six		1-All restoration maintained excellent anatomic form (100%) 2- only1-2 restorations showed slight slight contour deficiency overall anatomic form remains clinically acceptable. 3-One restoration in preheated group had Charlie score 4-All restorations remain alpha score 5-All restoration remains alpha score 6-no loss of anatomic form in any group	All restorations showed excellent anatomic form (mostly Alpha scores)	(*p* > 0.05)	Consistent

### Aesthetic properties

#### Marginal /surface discoloration

Only two studies [26, 30] (33.3%) revealed that conventional high-viscosity bulk-fill composites showed more marginal discoloration than other tested bulk-fill composite materials with a significant difference between them (*p* < 0.05). Conversely, the other three studies [25, 27, 28] reported no significant difference between the tested bulk-fill composite systems. However, Sajjan et al. [29] did not assess this parameter.

In Goda et al. [28] there was no evidence of marginal discoloration in all bulk-fill composite groups at different evaluation periods. Besides, the results demonstrated changes in surface discoloration among the four different tested materials (conventional, Sonicfill, preheated and injectable) with 85%,90%, 90% and 100% rates, respectively, with no significant difference between them (*p* > 0.05) at 2 years of follow-up. In Ibrahim et al., [27] all cases in all tested groups had Alpha score after 6 months. After 2-year follow-up, a one case in the conventional and 5-time preheated groups had bravo scores (*p* > 0.05). In Guney and Yazici [25], revealed a very low prevalence of marginal discoloration (6.5% of the flowable bulk-fill) with no measured difference between conventional and flowable bulk-fill restorations over a 2-year follow-up (*p* > 0.05).

In Morsy et al., [30] minor marginal discoloration was noticed only in the conventional group after a 1-year follow-up, as only 2 restorations showed Bravo scores (*p* > 0.05). After 18-month follow-up, marginal discoloration was detected in all tested groups: 6 restorations in the conventional group and only one restoration recorded in both other tested groups (preheated and Sonicfill) with a statistically significant difference between conventional versus (preheated and Sonicfill) groups (*p* < 0.05). Sonicfill and preheated groups had no difference (*p* > 0.05). In Elkady et al., [26] showed that all restorations performed by conventional or different preheated cycles retained Alpha scores at baseline. The conventional high-viscosity bulk-fill group showed more marginal discoloration at the one-year follow-up compared to the different preheated cycles groups. Hence, the conventional group showed a significant difference compared to the different preheated cycle groups (*p* < 0.05).

#### Color match

Five (83.3%) of the six included trials [25–27, 29, 30] evaluated color match. However, one study [26] revealed that conventional high-viscosity bulk-fill resin composite had a significant difference in comparison to the repeatedly preheated group, for ten times, as color mismatch was seen in 15 restorations of the conventional high-viscosity group and 9 ,4 restorations in the different preheating cycles groups with Alpha scores of 57.1%, 74.3%, and 88.6%, respectively. Another study [30], found that all tested (conventional, preheated, and Sonicfill) composite materials maintained color matching till 1-year follow-up, with only a slight decline after18-months; with no significant differences at all evaluation periods (*p* > 0.05). The other studies [25, 26, 29]showed no significant difference regarding this parameter. Goda et al. [28] did not evaluate the tested restorations regarding color match.

#### Surface texture

Four (66.6%) of included trials [25, 27–29] assessed surface texture, and all of them revealed no significant differences between various bulk-fill resin composite types (*p* > 0.05). Goda et al., [28] reported that all restorations in four tested materials (conventional, preheated, Sonifill and injectable) showed excellent surface luster. Ibrahim et al., [27] reported no significant differences in the surface texture between conventional and different preheated cycle restorations during various observational periods (*p* > 0.05). Sajjan et al., [29] reported that only one restoration in preheated group received Bravo score. Guney and Yazici [25], reported that 2 flowable bulk-fill restorations (6.5%) exhibited minimal surface roughness without statistical significant difference compared with conventional high-viscosity bulk-fill restorations after 2-years follow-up (*p* > 0.05). However, Morsy et al. [30] and Elkady et al. [26] did not use surface texture as a parameter for evaluation. Detailed aesthetic outcomes are summarized in Table 6.

**Table 6:** Synthesis matrix, statistical significance, consistency/ heterogeneity of aesthetic outcomes

Outcome	No. of Studies	Study (Author, Year	Finding	Summary finding	Statistical Significance	Consistency/ Heterogeneity
Marginal / Surface Discoloration		2-Morsy et al.[30] 3-Elkady et al. [26] 4-Ibrahim et al. [27] 5- Guney & Yazici [25] 6- Sajjan et al. [29]	1-No evidence of marginal discoloration in any group 2-Minor discoloration only in conventional group after 1 year; not significant. After 18-months, marginal discoloration was detected in all tested groups, with significant difference between conventional and other tested materials 3- conventional group showed more marginal discoloration compared to different preheated cycles groups after 1 years 4-All restorations had alpha scores at baseline; one restoration in the conventional group and one in the five-time preheated group received Bravo scores after two years (*p* > 0.05). 5-Very low prevalence of discoloration (6.5%), no significant difference 6-Not assessed	Conventional composites tended to show more marginal discoloration in 2 trials [26, 30] Others reported no significant differences among groups	Mixed	Moderate inconsistency
Color Match		2-Morsy et al. [30] 3-Elkady et al. [26] 4-Ibrahim et al. [27] 5- Guney & Yazici [25] 6- Sajjan et al. [29]	Did not evaluate color match 2-Acceptable match reported and maintained for 1 years with only a slight decline after18 months; with no significance differences at all evaluation periods 3- revealed that conventional had a significant difference in comparison to repeatedly preheated group for ten times 4-Similar color match during follow-up; no significant difference5-No significant difference in color match 6-No significant difference during recall evaluation	one study [26] revealed that conventional had significant difference in comparison to repeatedly preheated group for ten times others showed there was no significant difference regarding this parameter	Mixed:	Low–moderate heterogeneity
Surface Texture			2-Did not report surface texture 3-Not used surface texture as a parameter 4-No significant difference in surface texture Minimal roughness, no significant difference	All four trials reported excellent luster and smoothness	four trails reported (*p* > 0.05)	Consistent

### Biological properties

#### Postoperative sensitivity and vitality

All included studies showed that there was no significant difference between conventional high-viscosity and other types of bulk-fill composite systems (*p* > 0.05). There were no endodontic problems observed in any of the included trials except one restoration reported by Guney and Yazici [25]. 

In Morsy et al.‘s study [30], postoperative sensitivity was seen in only two cases (conventional and Sonicfill) at baseline, and it disappeared after six months, while no sensitivity was recorded in the preheated group at baseline and stayed stable during the study period. In Ibrahim et al. [27], one case in each evaluated group (conventional) and (different preheating- cycles) bulk-fill composite systems demonstrated a low incidence of tooth sensitivity. (4.8%) after 6 months. After two years follow-up, only (different preheating-cycle group) showed post-operative sensitivity (*n* = 1), but did not statistically differ from (conventional) high-viscosity bulk-fill composite systems (*p* > 0.05).

Goda et al. [28] demonstrated no sensitivity during different follow up periods. In Sajjan et al. [29] post-operative sensitivity was presented only in the preheated group (*n* = 1) at 6-month follow-up. No additional sensitivity was reported in both materials (preheated and Sonic) at 1-year recall .In the study by Guney and Yazici [25], patients reported minor postoperative hypersensitivity for 3 conventional bulk-fill restorations and 1 flowable bulk-fill restoration. One year later, one of three restorations in the conventional group received root canal treatment. The sensitivity completely disappeared from both materials after 2 years.

#### Secondary caries

No secondary caries was documented in any included study during the different follow-up periods. No significant difference between the conventional high-viscosity and other bulk-fill composite types and no significant difference between different bulk-fill composite types.

#### Periodontal and mucosal responses

According to the reviewed studies, only one study [28] reported excellent periodontal and mucosal response (100%) regarding periodontal and mucosal response with no significant difference between them (*p* > 0.05). However, other studies did not assess this parameter [25–27, 29, 30]. Synthesis matrix of Biological Outcome in Clinical Studies presented in Table 7.

**Table 7:** Synthesis matrix, statistical significance, consistency/ heterogeneity of biological outcomes

Outcome	No. of Studies		Findings	Statistical Significance	Consistency / Notes
Postoperative sensitivity	5 studies	1-Morsy et al. [30] 2-Ibrahim et al. [27] 3-Goda et al. [28] 4-Sajjan et al.[29] 5-Guney & Yazici. [25]	1-Sensitivity in conventional & sonic-fill at baseline, resolved after 6 months; no sensitivity in preheated group 2-One case in each group at 6 months (4.8%); after 2 years, one sensitivity case in preheated group; (*p* > 0.05). 3-No sensitivity reported in any group during follow-up 4-One case in preheated group at 6 months; none in either group at 1 year. 5-minor postoperative hypersensitivity occurred in three conventional and one flowable restoration; one conventional restoration required root canal treatment after one year, and symptoms resolved after two years	no significant differences (*p* > 0.05)	Consistent; no clinically relevant problems
Secondary caries	6 studies	All six included studies	*No secondary caries observed during follow-up in any group.	Not applicable	Fully consistent
Periodontal & mucosal response	1 study	Goda et al. [28]	Periodontal response (FDI)Excellent periodontal/mucosal scores (100%) excellent scores at 1–2 years	no significant differences (*p* > 0.05)	Not assessed in other studies [25–27, 29, 30]

## Discussion

Systematic reviews provide a comprehensive evaluation of available clinical evidence [31]. They are considered a valuable tool for assessing treatment outcomes [32]. Bulk-fill resin composite was introduced to address the problems associated with incremental technique [25, 33, 34]. Bulk-fill resin composite was introduced with different viscosities: high- and low- viscosity bulk-fill resin composite [35, 36]. Furthermore, manufacturers have introduced several modifications, leading to notable differences in the clinical placement methods of various bulk-fill composites, which can impact their clinical performance [37]. Thus, the purpose of conducting this systematic review was to compare the clinical performance of various bulk-fill resin composite types including sonic-activated, preheated, and/or flowable bulk-fill composite versus conventional high-viscosity bulk-fill composite in any cavity design.

This systematic review included previously conducted clinical studies with at least two different types of bulk-fill resin composite. clinical studies that evaluated clinical performance of a single type or category of bulk-fill resin composite restorative systems were excluded [38]. As this review aimed to compare different categories of bulk-fill composite to each other. Furthermore, clinical studies comparing bulk-fill resin composite versus incremental ones were also excluded. These exclusion and inclusion criteria explain why only the limited number of included clinical studies in this review. Studies conducted in the last 10 years were also included because of the development and availability of bulk-fill composite materials. Studies performed on endodontically treated teeth were excluded too due to loss of vitality affecting the biomechanical behavior of teeth, leading to failure.

All studies recorded a high risk of bias due to the impossibility of blinding operators. As they needed to be aware of the restorative material during placement. This consistently high risk of performance bias represents a significant methodological limitation and may influence the certainty of the overall findings.

According to the clinical studies included in this review, various types of bulk-fill composite, either sonic-activated, preheated, or flowable, demonstrated good clinical performance compared to conventional high-viscosity bulk-fill resin composite for the specific clinical parameters: retention, postoperative sensitivity, secondary caries, and anatomic form. However, the evidence regarding marginal adaptation and marginal discoloration remain debatable. No clinically relevant differences were observed between sonic-activated and preheated bulk-fill composites across the evaluated clinical parameters.

### Retention

Retention outcomes showed that no fractures occurred in any of the bulk-fill composite materials, confirming their clinical longevity [39]. This may be related to the sufficient mechanical properties of the bulk-fill composite materials [40, 41], adequate use of adhesive technique and proper cavity design, as all included studies used either class I or class II cavities. This was in agreement with the results of a Sengupta ‘s systematic review. Only one preheated restoration was lost in one study [29], which may be due to a technical error during composite placement.

### Anatomic form

Anatomic form remains constant across all bulk-fill composite types. This may be attributed to filler type and composition. Also, the clinically acceptable proximal contact observed in the included studies [26–28] might be attributed to the use of pre-contoured sectional bands with a separation ring.

### Marginal adaptation

Marginal defects may degrade the restoration’s seal, causing restoration repair or replacement. Marginal adaptation is affected by different factors including resin composite viscosity, long-term degradation of the bonding system and polymerization shrinkage of the composite material utilized [42]. Three of the included studies showed no significant difference between conventional high-viscosity and other types of bulk-fill resin composite, this may be due to the conventional high-viscosity bulk-fill composite used. In the first study [27] the conventional bulk-fill composite used was a low shrinkage material (Xtra fil) with a filler content that exhibited minor polymerization shrinkage of 1.7%, leading to satisfactory marginal adaptation in all observation periods. The second study [28] used a conventional bulk-fill composite containing a modified UDMA, AFM and DDMA in matrix It is responsible for low polymerization shrinkage and good marginal adaptation [43]. The third study [25] used conventional high-viscosity and flowable bulk-fill resin composite with conventional layered composite with the same level of polymerization shrinkage. This resulted in the same degree in marginal adaptation for both groups.

The same conventional high-viscosity bulk-fill composite with modified matrix used by Goda et al. [28] was used in another study [30], showing adequate marginal adaptation for one year. However, degradation in the marginal seal was shown after 18 months, with a significant difference compared with other intervention groups. This may be due to degradation of the single bond universal adhesive with time. This degradation was observed by SEM, which was used in this study; the naked eye could not detect any defects. One study [26] reported that conventional high-viscosity bulk-fill composite had more marginal defects than other intervention groups from baseline, due to Polymerization shrinkage of the conventional high-viscosity bulk-fill composite that generates stress at the tooth-restoration interface. Hence, a significant marginal gap was observed [44, 45]. 

On the other hand, all intervention bulk-fill groups either sonic-activated, preheated, and/or flowable showed comparable results. The excellent marginal seal of these materials may be related to the technologies used to reduce viscosity. As, the sonic device provides vibration through an air-driven handpiece, reducing the composite viscosity by approximately 84%, thereby improving marginal adaptation [29]. The results are in agreement with Akalin et al. [13] Additionally, the good marginal adaptation of preheated resin composite is due to decreased viscosity and improved physical properties [46]. Similarly, flowable composite have lower viscosity, which enhances their marginal adaptation to cavity walls [47]. 

### Marginal discoloration

Only two studies [26, 30] reported significant differences between conventional high-viscosity and other types of bulk-fill resin composite. In one study [26], marginal discoloration was noticed only in the conventional high-viscosity group due to inadequate marginal adaptation [48]. In contrast, the preheated intervention groups, demonstrated improved marginal adaptation because of decreased composite viscosity, thereby reducing the margins’ susceptibility to stain entrapment. In another study [30], marginal discoloration was observed in the conventional high-viscosity group and was attributed to inadequate marginal adaptation in conventional high-viscosity bulk-fill composite leading to significant discoloration compared to other intervention groups (preheated and sonic activated). After an 18-month follow-up, marginal discoloration was detected in all tested groups. This may be attributed to the use of a self-etch adhesive without prior phosphoric acid etching [39]. This finding is in agreement with a pervious systematic review [19]. In comparison, minor discolorations without a significant difference were observed in the Sonicfill and preheated composite as their viscosity was decreased by sonic vibration or preheating; resulting in better adaptation to the cavity walls.

Another study reported [28] external surface discolorations with no significant differences. This discoloration may be attributed to the patients’ consumption of acidic beverages. This finding is in agreement with a laboratory study [49]. On the other hand, the injectable bulk-fill composite showed no superficial surface discoloration. This is may be attributed to its ultra-fine barium filler particles that are small, uniformly distributed, and densely packed, it did not exhibit any surface discoloration [50]. 

### Color match

One study [26] demonstrated that the conventional high-viscosity bulk-fill composite had significantly inferior color match than the other bulk-fill composite types after a 1-year follow-up. The decline in color matching of the conventional high-viscosity bulk-fill composite was due to the presence of voids and stains. These findings may be explained by the incremental placement method and the inferior handling properties of the used material.

### Surface texture

The maintained surface texture after different follow-up periods may be attributed to the use of an adequate polishing protocol [51]. Additionally, Bisphenol A-glycidyl methacrylate (Bis-GMA) is the most commonly used resin monomer in bulk-fill resin composite materials. It is less flexible and more rigid [52]. 

### Post-operative sensitivity and vitality

Post-operative sensitivity was not observed in most of the included studies, regardless of the restorative material used. Such a clinical finding may be attributed to the operator’s skills in performing all restorative procedures, including the use of sharp cutting burs under copious amount of water and proper isolation [53]. It may also be attributed to the use of a calcium hydroxide liner in deep areas, which were sealed with glass ionomer cement, and the omission of a separate phosphoric acid etching step in one study [30]. Endodontic complications were not observed in any included study except for one restoration in the conventional high-viscosity bulk-fill group reported by Guney and Yazici [25]. This restoration required root canal treatment because of persistent postoperative sensitivity, which the authors suggested could be related to the patient’s low pain perception.

### Secondary caries

No secondary caries was observed in any of the included studies. This could be attributable to the selection of patients with good oral hygiene and operators’ skills in caries excavation [54]. Moreover, it could be attributable to the use of rubber dam isolation in the majority of the included studies [55]. 

### Study limitations

This systematic review has several limitations that must be considered when interpreting the findings:

1. Heterogeneity in study design: The included studies used both parallel [26-28] and split-mouth designs [25, 29, 30]. While split-mouth studies allow direct comparison within the same patient, parallel studies compare different patients, introducing variability in factors such as oral hygiene, salivary flow, and masticatory forces [56]. This variation may affect restoration performance and complicates the pooling of results, as differences may be due to patient variability rather than material performance.
2. Differences in clinical evaluation criteria: Some studies used the FDI criteria, while others used the USPHS system. The FDI system is more sensitive and can detect subtle changes in marginal integrity, surface luster, and wear. In contrast, the USPHS system may overlook minor defects. For example, a restoration with slight marginal discoloration might be detected by the FDI criteria but considered acceptable by the USPHS system, leading to inconsistent outcome reporting.
3. Methodological limitations and risk of bias: all included studies had high risk of performance bias because operator blinding was not feasible during restorative procedures. This methodological limitation may have influenced the reported outcomes.
4. Variation in follow-up durations: Follow-up periods differed between studies, with some assessing restorations for only 6-12 months. Short-term follow-ups may fail to capture long-term failures such as wear, fracture, or marginal deterioration, which are critical for evaluating the durability of bulk-fill composites.
5. Incomplete reporting of clinical parameters: Certain clinical parameters, including surface luster, fracture/integrity, wear, and proximal contact, were not consistently reported. For instance, missing data on wear prevented the assessment of functional longevity, while unreported proximal contact may mask potential clinical problems as food impaction or secondary caries.
6. Limited geographic representation: Most studies were conducted in Egypt and Turkey, potentially limiting the generalizability of the finding. Regional differences in diet, oral hygiene practices, and clinical techniques may affect restoration performance, meaning that the findings may not be applicable to populations in other countries.

### Clinical implications

The evidence suggests that different bulk-fill insertion techniques, including sonic-activated, preheated, and flowable, provide excellent clinical outcomes in terms of functional, aesthetic, and biological performance. For dental practitioners, these alternative techniques can be confidently considered for routine clinical use as reliable restorative options.

## Conclusion

This systematic review suggests that different bulk-fill resin composites demonstrate comparable outcomes across functional and biological parameters. However, the current evidence is limited and insufficient to draw definitive conclusions regarding marginal adaptation and marginal discoloration. Further long-term, well-designed randomized controlled trials with standardized methodologies are needed to confirm these observations and strengthen the reliability of the findings.

## Supplementary Information

Supplementary Material 1.

Supplementary Material 2.

## Data Availability

The data sets used and/or analysed during the current study available from the corresponding author on reasonable request.
