# Supplementary material for: Clinical performance of different types of posterior bulk-fill resin composite restorations in permanent teeth: a systematic review
Source: BMC Oral Health. 2026 Oct 26;26:2100. doi: 10.1186/s12903-026-10017-x (PMC13700169; doi:10.1186/s12903-026-10017-x)
Supplement: Supplementary file 1 [file 12903_2026_10017_MOESM1_ESM.pdf]

NIHR | National Institute for Health and Care Research

PROSPERO
International prospective register of systematic reviews

Clinical performance of different types of Bulk-fill resin composites: A systematic review

Asmaa Saad, Youniss Harp, Hamdi Hamama, Salah Mahmoud

To enable PROSPERO to focus on COVID-19 submissions, this registration record has undergone basic automated checks for eligibility and is published exactly as submitted. PROSPERO has never provided peer review, and usual checking by the PROSPERO team does not endorse content. Therefore, automatically published records should be treated as any other PROSPERO registration. Further detail is provided here.

Citation
Asmaa Saad, Youniss Harp, Hamdi Hamama, Salah Mahmoud. Clinical performance of different types of Bulk-fill resin composites: A systematic review. PROSPERO 2024 Available from https://www.crd.york.ac.uk/PROSPERO/view/CRD42025640119

REVIEW TITLE AND BASIC DETAILS

Review title
Clinical performance of different types of Bulk-fill resin composites: A systematic review

Review objectives
Do different types of bulk-fill composites have a well clinical performance in randomized clinical trials?

SEARCHING AND SCREENING

Searches
The National Library of Medicine (MEDLINE/PubMed) and ScienceDirect 2015-2025 / English

Study design
randomized clinical trials.

ELIGIBILITY CRITERIA

Condition or domain being studied
evaluate the clinical performance of different types of bulk-fill composite and compare them to each other regarding esthetic, biologic and functional properties

Population

Participants with permanent posterior teeth restored using direct composite restorations.

Intervention(s) or exposure(s)

Posterior teeth restored with other types of bulk fill composite either flowable BF, heated body BF or/and sonic activated BF.

Comparator(s) or control(s)

Posterior teeth received conventional high viscous bulk fill composite restorations.

OUTCOMES TO BE ANALYSED

Main outcomes

clinical performance of different bulk fill composite restorations regarding esthetic, biological and functional aspects using FDI or USPHS criteria.

Additional outcomes

None

DATA COLLECTION PROCESS

Data extraction (selection and coding)

The reviewer (A.S.) will extract data and will assess it is eligibility and risk of bias. After the data will tabulated and listed, a second reviewer (H.H.) will confirm the extracted data and risk of bias assessments. A third reviewer (S.H.) will consulted to settle any disagreements that emerged. Studies will chosen after three steps title , abstract then full text analysis

Risk of bias (quality) assessment

using the modified Cochrane Collaboration Risk of Bias Tool which included five domains: selection bias (randomization, allocation concealment), performance bias (blinding of participants and operators), detection bias (blinding of outcome assessment), attrition bias (loss to follow-up and missing values or participants), and reporting bias (unclear withdrawals or reported outcomes).

PLANNED DATA SYNTHESIS

Strategy for data synthesis

I not know if metanalysis will be made or not yet.
if there is heterogenicity in results , metanalysis can not made

Analysis of subgroups or subsets

selected studies will be get to each reviewer to confirm eligibility

REVIEW AFFILIATION, FUNDING AND PEER REVIEW

Review team members

- Dr Asmaa Saad, Conservative Dentistry Department, Faculty Of Dentistry, Mansoura University
- Dr Youniss Harp, Conservative Dentistry Department, Faculty Of Dentistry, Mansoura University
- Professor Hamdi Hamama, Conservative Dentistry Department, Faculty Of Dentistry, Mansoura University
- Professor Salah Mahmoud, Conservative Dentistry Department, Faculty Of Dentistry, Mansoura University

Review affiliation

Conservative Dentistry Department, Faculty Of Dentistry, Mansoura University

Funding source

Not applicable

Named contact

Asmaa Saad. Egypt/ Mansoura
dr.asmaa.saad266@gmail.com

TIMELINE OF THE REVIEW

Review timeline

Start date: 15 October 2024. End date: 01 April 2025

Date of first submission to PROSPERO

23 January 2025

Date of registration in PROSPERO

05 February 2025

CURRENT REVIEW STAGE

Publication of review results

The intention is not to publish the review once completed.

Stage of the review at this submission

Review stage	Started	Completed
Pilot work	✓	✓
Formal searching/study identification	✓	✓
Screening search results against inclusion criteria	✓	✓
Data extraction or receipt of IP		
Risk of bias/quality assessment		
Data synthesis		

Review status

The review is currently planned or ongoing.

ADDITIONAL INFORMATION

PROSPERO version history

- Version 1.1 published on 05 Feb 2025
- Version 1.0 published on 05 Feb 2025

Review conflict of interest

None known

Country

Egypt

Disclaimer

The content of this record displays the information provided by the review team. PROSPERO does not peer review registration records or endorse their content.

PROSPERO accepts and posts the information provided in good faith; responsibility for record content rests with the review team. The owner of this record has affirmed that the information provided is truthful and that they understand that deliberate provision of inaccurate information may be construed as scientific misconduct.

PROSPERO does not accept any liability for the content provided in this record or for its use. Readers use the information provided in this record at their own risk.

Any enquiries about the record should be referred to the named review contact
