# Supplementary material for: Clinical performance of different types of posterior bulk-fill resin composite restorations in permanent teeth: a systematic review
Source: BMC Oral Health. 2026 Oct 26;26:2100. doi: 10.1186/s12903-026-10017-x (PMC13700169; doi:10.1186/s12903-026-10017-x)
Supplement: Supplementary file 2 [file 12903_2026_10017_MOESM2_ESM.docx]

**Manuscript Title:** Clinical performance of different bulk-fill resin composites in posterior teeth: A systematic review
**Registration Number:** PROSPERO (CRD42025640119)

| **Section/Topic** | **Item #** | **Checklist item** | **Location in Manuscript** |
| --- | --- | --- | --- |
| **Title** | 1 | Identify the report as a systematic review | Title page |
| **Abstract** | 2 | Structured summary including objectives, eligibility criteria, methods, results, and conclusions | Abstract |
| **Introduction** | 3 | Rationale for the review | Page 4 |
|  | 4 | Objectives of the review | Page 4 |
| **Methods** | 5 | Eligibility criteria | Pages 5–6 |
|  | 6 | Information sources | Page 6 |
|  | 7 | Search strategy | Page 6 |
|  | 8 | Selection process | Page 8; Figure 1 |
|  | 9 | Data collection process | Page 6 |
|  | 10 | Data items (PICO(S), outcomes) | Pages 4–5 |
|  | 11 | Risk of bias assessment (RoB 2) | Page 5 |
|  | 12 | Effect measures | Not applicable |
|  | 13 | Synthesis methods | Page 7 |
|  | 14 | Reporting bias assessment | Not applicable |
|  | 15 | Certainty assessment | Not applicable |
| **Results** | 16 | Study selection (flow diagram) | Page 8; Figure 1 |
|  | 16a | Records identified = 1041 | Figure 1 |
|  | 16b | Records screened = 800 | Figure 1 |
|  | 16c | Records after title screening = 233 | Figure 1 |
|  | 16d | Full-text articles assessed = 33 | Figure 1 |
|  | 16e | Studies included = 6 RCTs | Figure 1 |
|  | 17 | Study characteristics | Tables 2 & 3 |
|  | 18 | Risk of bias in studies | Table 1; Figures 2 & 3 |
|  | 19 | Results of individual studies | Tables + text |
|  | 20 | Results of syntheses | Pages 6–8 |
|  | 21 | Reporting biases | Not applicable |
|  | 22 | Certainty of evidence | Not applicable |
| **Discussion** | 23 | Interpretation of results | Pages 33—37 |
|  | 24 | Limitations of included studies | Page 37-38 |
|  | 25 | Limitations of review process | Page 37-38 |
|  | 26 | Implications for practice and research | Page 39 |
| **Other Information** | 27 | Registration and protocol | Page 4 (PROSPERO) |
|  | 28 | Funding/support | Acknowledgments |
|  | 29 | Competing interests | Declarations |
|  | 30 | Availability of data | Supplementary materials |
